# Intramolecular Lactones of Sialic Acids

**DOI:** 10.3390/ijms21218098

**Published:** 2020-10-30

**Authors:** Paola Rota, Paolo La Rocca, Pietro Allevi, Carlo Pappone, Luigi Anastasia

**Affiliations:** 1 Department of Biomedical, Surgical and Dental Sciences, University of Milan, 20133 Milan, Italy; pietro.allevi@unimi.it; 2 Department of Biomedical Sciences for Health, University of Milan, 20133 Milan, Italy; paolo.larocca@unimi.it; 3 Arrhythmology Department, IRCCS Policlinico San Donato, 20097 San Donato Milanese, Milan, Italy; carlo.pappone@grupposandonato.it; 4 Faculty of Medicine, University of Vita-Salute San Raffaele, 20132 Milan, Italy; 5 Laboratory of Stem Cells for Tissue Engineering, IRCCS Policlinico San Donato, 20097 San Donato Milanese, Milan, Italy

**Keywords:** lactone, sialic acid, biomarker, heptafluoro derivatives, tumor

## Abstract

The so-called “*sialo-chemical-biology*” has become an attractive research area, as an increasing number of natural products containing a sialic acid moiety have been shown to play important roles in biological, pathological, and immunological processes. The intramolecular lactones of sialic acids are a subclass from this crucial family that could have central functions in the discrimination of physiological and pathological conditions. In this review, we report an in-depth analysis of the synthetic achievements in the preparation of the intramolecular lactones of sialic acids (1,4-, 1,7- and γ-lactones), in their free and/or protected form. In particular, recent advances in the synthesis of the 1,7-lactones have allowed the preparation of key sialic acid derivatives. These compounds could be used as authentic reference standards for their correct determination in biological samples, thus overcoming some of the limitations of the previous analytical procedures.

## 1. Introduction

**Figure 1:**
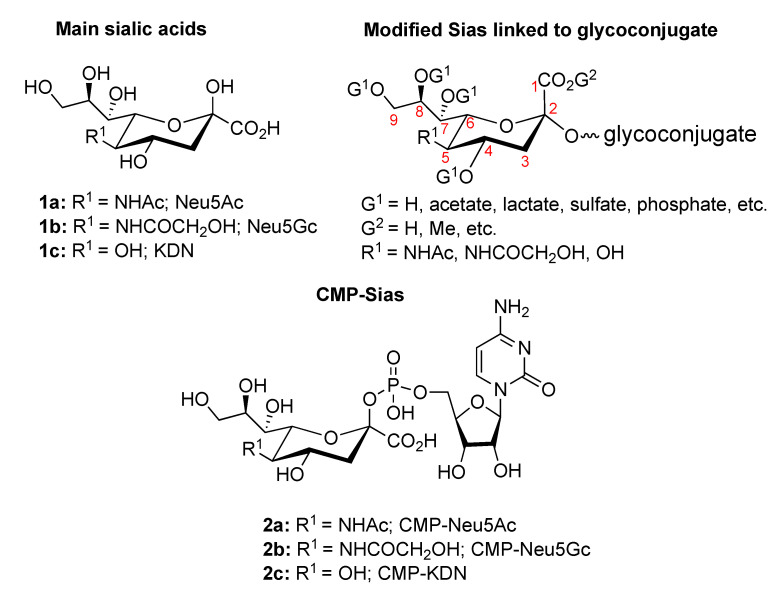
Representation of the three principal sialic acids (Neu5Ac **1a**, Neu5Gc **1b**, and KDN **1c**), the general structure of modified Sias linked to the glycoconjugate chain, and the three cytidine mononucleotide Sias (CMP)-Sias (**2a–c**).

Sialic acids (Sias) are a family of nine-carbon α-keto aldonic acids, including both the derivatives of the progenitor neuraminic acid (Neu, 5-amino-3,5-dideoxy-D-*glycero*-D-*galacto*-2-nonulosonic acid), as the *N*-acetylneuraminic acid (Neu5Ac) **1a** and the *N*-glycolylneuraminic acid (Neu5Gc) **1b**, and those of the later discovered 3-deoxy-D-*glycero*-D-*galacto*-2-nonulosonic acid or 2-keto-3-deoxy-D-*glycero*-D-*galacto*-nononic acid (KDN **1c**), bearing a hydroxyl group in place of the amino group at the C5 position (Figure 1) [1,2,3,4,5,6,7,8].

These carbohydrates possess several unique characteristics. First, they usually occupy the distal end of the glycoconjugate glycan chains to which they are attached, which is the ideal location to mediate the interaction of the cell with the external environment, including other cells and pathogens. Therefore, they play important roles in physiological, pathological, and immunological processes [1,2,4,5,6]. Second, Sias-containing natural compounds are often susceptible to structural biological modifications, generating a family of more than eighty distinct members [1,4,5]. Third, due to their peculiar structures, they present unique biochemical characteristics as compared to common monosaccharides [1,3,4,7]. Indeed, they are biosynthesized by condensation of a neutral six-carbon unit sugar with pyruvate, and they are activated in the form of CMP- Sias (i.e., **2a–c**) [1,4].

**Figure 2:**
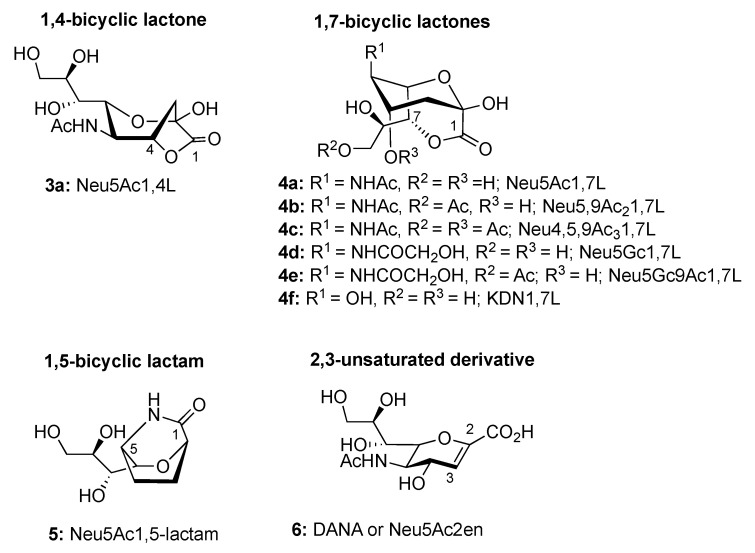
Representation of the 1,4-bicyclic lactone **3a**, the six 1,7-bicyclic lactones **4a–f**, the 1,5-bicyclic lactame **5** and the 2,3-unsaturated derivative DANA **6**.

All the currently recognized sialic acids are biosynthetic derivatives of one of the two main family members, Neu5Ac **1a** and KDN **1c** (Figure 1). This is true also for the third most important derivative of the family, Neu5Gc **1b**, which is biosynthetically derived from Neu5Ac-CMP **2a** through CMP-Neu5Ac hydroxylase catalysis [9,10,11,12,13,14,15]. The structural diversity of sialic acids is primarily generated by combinations of different substitutions at the C5 position or by replacement of the hydroxyl groups at the C4, C7, C8, and C9 positions with either acetate, lactate, sulfate, phosphate- or methyl-ethers (Figure 1) [1,4,7]. Moreover, other Sias derivatives, obtained by intra-lactonization of the carboxylate at C1 with different internal hydroxylic functions, have also been described, including compounds **3a** [16] and **4a–f** [17,18,19,20,21] (Figure 2). Furthermore, the large family of Sias also comprises a group of intramolecular 1,5-lactams, which are derived from compound **5** [22,23,24,25,26] and 2,3-unsaturated derivatives (2-deoxy-2,3-dehydro *N*-acetylneuraminic acid, DANA **6**) [27,28,29,30,31,32,33,34,35].

Remarkably, the lactones of Sias have been related to various pathologies, both when they are formed with other carbohydrates that precede them in the same glycan chain of glycoconjugates [36,37,38,39,40,41,42,43,44,45,46,47,48] and when they are formed by intramolecular lactonization [16,17,18,19,20,21]. In particular, this review will focus on the intramolecular monocycles and bicyclic lactones, clarifying the current state of the art on these molecules at the interface of chemistry and biology. Their relevance in various pathologies [49,50,51,52,53,54,55,56,57,58,59,60,61,62,63,64,65] has led to the synthesis and the development of an analytical technique to detect them in biological media [60]. However, we need to take into account that the recent discovery of several pitfalls [66] in the previous analytical method [60] suggests that the genuine presence of these molecules in biological matrices should be reassessed.

## 2. Overview of the Sias Intramolecular Lactones: Synthesis and Rearrangement Mechanism

### 2.1. Synthesis of 1,4- and 1,7-bicyclic Lactones of Neu5Ac

**Scheme 1:**
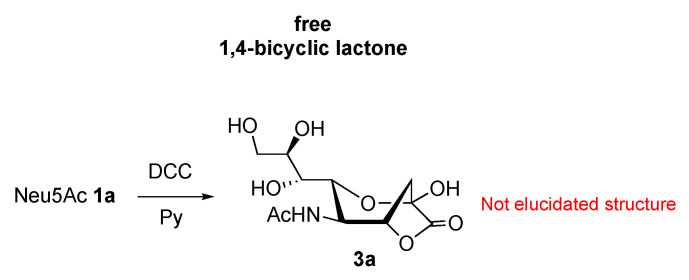
Synthesis of the free 1,4-bicyclic lactone **3a** starting from Neu5Ac **1a** [16].

The first synthetic route to achieve free or protected intramolecular bicyclic lactones of Neu5Ac was published by Dereviskaya et al. in 1966 [16], affording the 1,4-bicyclic lactone **3a**. The authors claimed to obtain this molecule by treating Neu5Ac **1a** with *N*,*N*′-dicyclohexylcarbodiimide (DCC), as a condensing agent, in pyridine followed by electrophoretic purification (Scheme 1); however, the structure of the obtained lactone was not sufficiently supported by physico-chemical data. In fact, after this initial report, the formation of this 1,4-bicyclic lactone **3a** has never been observed again, nor could the compound be detected in any biological sample.

**Scheme 2:**
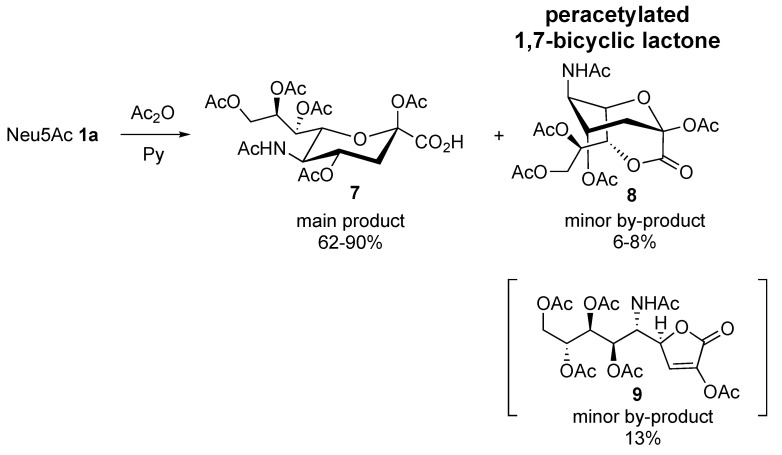
Synthesis of the peracetylated 1,7-bicyclic lactone **8** as by-product of Neu5Ac **1a** classical acetylation [71,72,73].

In the 1980s and 1990s, two independent research groups (Ogura’s and Gervay’s teams) attempted to synthesize both the intramolecular 1,4- and 1,7-bicyclic lactones, obtaining them only in their protected form [67,68,69,70,71]. In particular, Ogura’s group reported seeding results in the synthesis, structure elucidation, as well as in the mechanism of formation of these protected bicyclic lactones [71]. Initially, they reported, almost concurrently with Kirchner et al. [72], that the peracetylation of Neu5Ac **1a** also afforded, together with the desired peracetylated compound **7**, the 1,7-lactone of Neu5Ac **8** as a minor by-product (Scheme 2). Its structure was previously incorrectly attributed, by Khorlin et al. [73], to the 5-acetamido-2,6,7,8,9-penta-*O*-acetyl-3,5-dideoxy-D-*glycero*-D-*galacto*-2-noneno-1,4-lactone **9**, a protected monocyclic Neu5Ac 1,4-lactone, known as γ-lactone (see Section 2.3). The correct structure of the peracetylated 1,7-lactone of Neu5Ac **8** was clarified by combining nuclear magnetic resonance (NMR) analysis, mass spectrometry (MS) and X-ray crystal analysis [71,72].

**Scheme 3:**
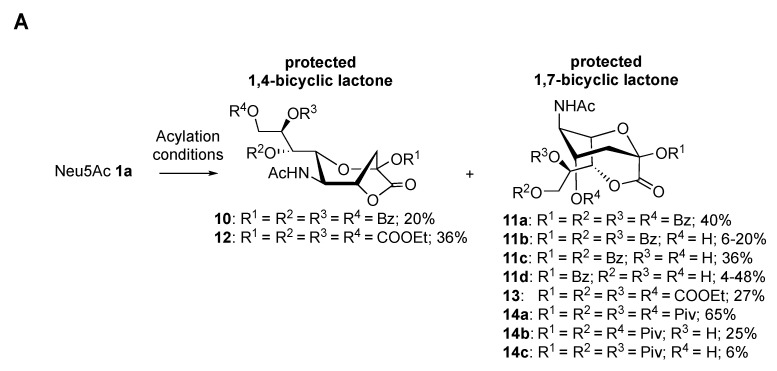

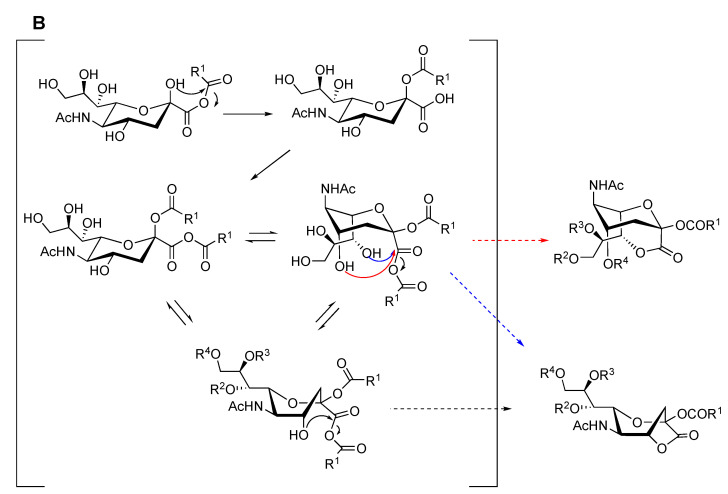
(**A**) Synthesis of the protected 1,4-bicyclic lactones **10**, **12** and the 1,7-bicyclic lactones **11a–d**, **13** and **14a–c** starting from Neu5Ac **1a** [70]; (**B**) proposed mechanism of intramolecular lactonization [70].

The peracetylated 1,7-lactone of Neu5Ac **8** was subsequently identified by Gervay’s group [74] as a minor by-product (2% yield) during the synthesis of DANA **6**, a key precursor of many antiviral drugs. Moreover, Ogura’s group reported an in-depth study on protected 1,4- and 1,7-bicyclic lactones (Scheme 3) [70]. They isolated these lactones, protected at the hydroxylic functions, by subjecting Neu5Ac **1a** to acylating conditions (benzoyl chloride or benzoyl anhydride, pivaloyl chloride and ethyl chloroformate). Indeed, mixtures of compounds **10** and **11a–d** were obtained using benzoyl agents, while compounds **12** and **13** were synthesized using ethyl chloroformate or **14a–c**, using pivaloyl chloride (Scheme 3A). The correct structure of these bicyclic lactones was elucidated by ^1^H-NMR spectroscopy. Noteworthily, they proposed a fascinating mechanism of intramolecular lactonization (Scheme 3B), where a mixed anhydride on the carboxylic function is initially formed, and it is successively attacked by the 2-hydroxylic group, giving the 2-acylated derivative. Subsequently, the carboxylic function is again activated, leading to a conformational rearrangement. Therefore, the 4- or 7-hydroxylic group can promote an internal 1,4- or 1,7-lactonization; this second possibility is favored by the presence of sterically bulky substituents. Finally, the partial or total acylation of other hydroxylic functions occurs [70].

**Scheme 4:**
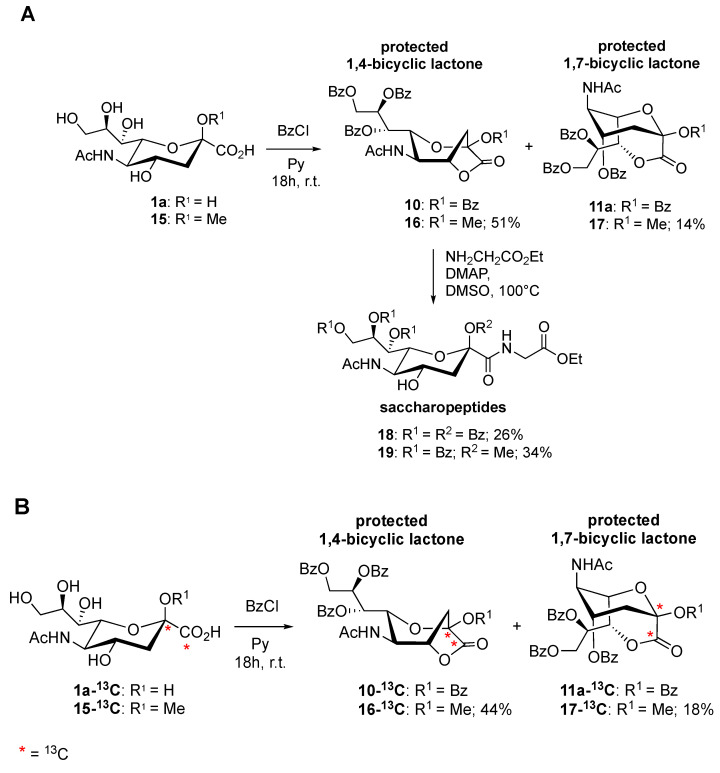
(**A**) Synthesis of the saccharopeptides **18** and **19** via the protected 1,4-bicyclic lactones **10** and **16** [67]; (**B**) synthesis of the protected ^13^C-labeled 1,4-bicyclic lactones **10-^13^C** and **16-^13^C** and the 1,7-bicyclic lactones **11a-^13^C** and **17-^13^C** [68].

In the 1990s, in light of Ogura’s work, Gervay’s group attempted the synthesis of these bicyclic lactones, but they obtained them only in the protected form (Scheme 4). In particular, they proposed [67] the use of the 1,4-protected compounds as starting material to synthesize a new class of molecules, the amino acid-NeuAc saccharopeptides (Scheme 4A). For this purpose, starting from Neu5Ac **1a,** or from its 2-*O*-methyl derivative **15**, they synthesized mixtures of 1,4- and 1,7-lactones, **10** and **11a**, or **16** and **17**, respectively. Then, lactones **10** and **16** were treated with protected glycine to give the desired saccharopeptides **18** or **19**. Importantly, this synthetic strategy also allowed the obtainment of several sialyl donors. A second application that was proposed [68] concerned the use of protected ^13^C-labeled intramolecular 1,4- and 1,7-lactones, **10-^13^C** and **11a-^13^C** or **16-^13^C** and **17-^13^C**, obtained from Sias **1a-^13^C** and **15-^13^C**, respectively. They are suitable model compounds to study more complex sialyllactones by the isotope edited NMR spectroscopy technique (Scheme 4B). Notably, the authors concluded that further investigations would have been necessary to confirm the applicability of the proposed method also on the unprotected 1,4- or 1,7-lactones, as they failed to obtain them in free form. Interestingly, Gervay’s group performed a computational study [69] to evaluate whether the sialyllactones, or their derivatives, could be a valid scaffold in the design of high-affinity neuraminidase inhibitors. For this purpose, they used virtual free 1,4- and 1,7-bicyclic lactones to compare the ability of different computational methods to reproduce the NMR constant coupling of these molecules.

**Scheme 5:**
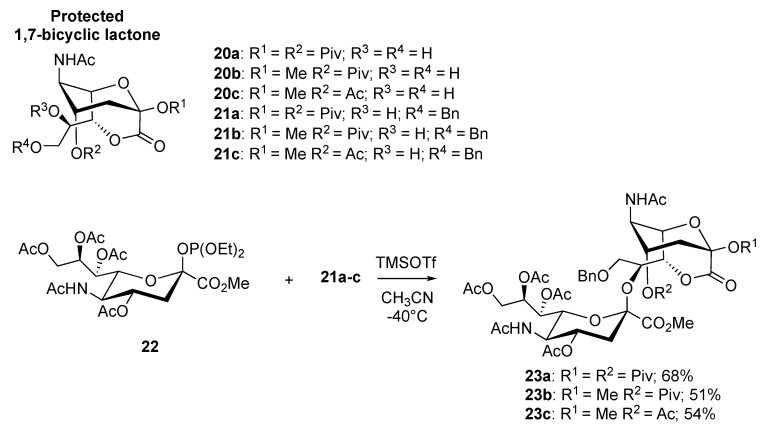
Protected 1,7-byciclic lactones **20a–c** and **21a–c** and synthesis of the β-2,8-unnatural saccharides **23a–c** [75].

Richard R. Schmidt’s group reported a biologically interesting study concerning the synthesis of some protected Neu5Ac 1,7-bicyclic lactones (**20a–c** and **21a–c**; Scheme 5) [75]. They used the lactones **21a–c**, as sialyl acceptors, and the phosphite **22**, as a donor, for the synthesis of β-2,8-unnatural saccharides **23a–c**. This synthetic strategy represented a novel and interesting approach for the preparation of unusual gangliosides starting from these 1,7-lactonic compounds.

**Scheme 6:**
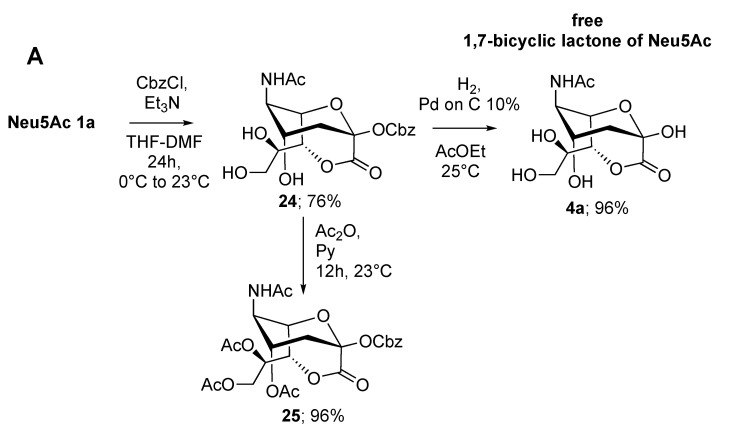

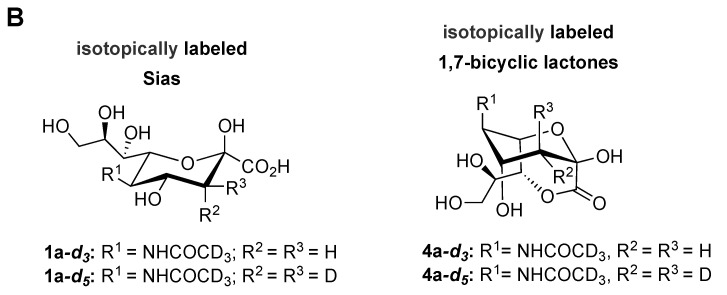
(**A**) Synthesis of the free 1,7-bicyclic lactone of Neu5Ac **4a** starting from Neu5Ac **1a** [17]; (**B**) isotopically labeled 1,7-byciclic lactones **4a-*d*_3_** and **4a-*d*_5_** derived from the corresponding Sias **1a-*d*_3_** and **1a-*d*_5_** [18].

Overall, these seminal studies reached several important goals, as they: (i) established synthetic routes for the synthesis of protected 1,4- and 1,7- lactones, yet still in as mixtures; (ii) elucidated their correct structures, which was not obvious; (iii) clarified the molecular mechanism leading to their formation, which was also intricate; (iv) showed their application in the biological field. However, the main limit of these studies was that they all failed in delivering the free bicyclic lactones, as only the corresponding protected derivatives could be obtained. Indeed, to date, the synthesis of the free 1,4-bicyclic lactone **3a** remains elusive. On the other hand, the synthesis of the free 1,7-lactones (**4a**, **4e**, and **4f**) was accomplished by Allevi’s group in the 2000s [17,18,19,20,21,66,76], starting from Neu5Ac, Neu5Gc, and KDN, respectively (for the synthesis of 1,7-lactones of Neu5Gc and KDN, **4e** and **4f**, see Section 2.2). Actually, the effective idea for the synthesis of lactone **4a** was simply the use of a bulky and easily removable acylating agent. For this purpose, Neu5Ac **1a** was treated with benzyl chloroformate in a dimethylformamide–tetrahydrofuran (DMF–THF) mixture containing triethylamine, affording the 1,7-lactone **24**, exclusively protected at the anomeric hydroxylic function, as reported in Scheme 6. The synthesis of the desired free *N*-acetylneuraminic acid 1,7-lactone (Neu5Ac1,7L) **4a** [77] was accomplished by hydrogenolysis of the benzyloxy carbonyl function of compound **24** in anhydrous ethyl acetate. The structure of the intermediate **24** was attributed by performing a complete NMR analysis and its synthetic conversion into the peracetylated derivative **25** [17]. Moreover, the low stability of this 1,7-lactone **4a**, under neutral or acidic aqueous solutions, was also suggested [17,18,19,20,21,66,78] (see Section 3.4). Notably, in this way, they could validate the mechanism of the intramolecular lactonization, which was previously hypothesized by Ogura and co-workers [70].

Furthermore, the same authors also synthesized the deuterated isotopologues of the 1,7-lactone **4a** for its use as an authentic internal standard for high-performance liquid chromatography (HPLC) and GC analyses, coupled with mass spectrometry [18]. The isotopically labeled lactones **4a-*d_3_***, **4a**-***d_5_*** have been synthesized, under similar reaction conditions as for **4a**, starting from the isotopically labeled Sias **1a-*d_3_***, **1a**-***d_5_***, which were labeled with deuterium at the amido group alone or both at the amido-function and the C3-position.

### 2.2. Synthesis of 1,4- and 1,7-bicyclic Lactones of Neu5Gc and KDN

**Scheme 7:**
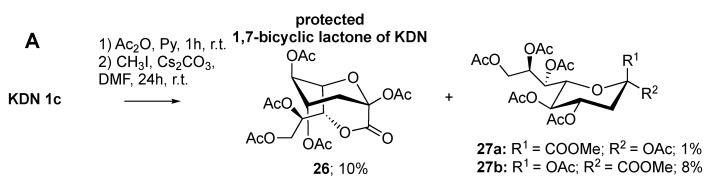

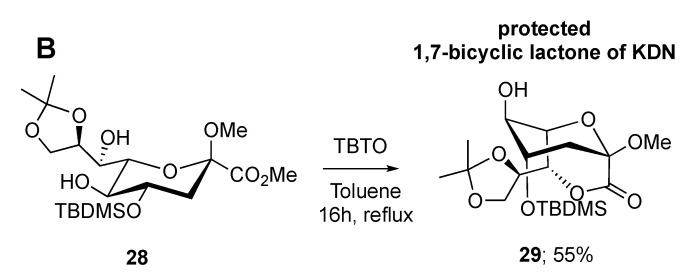
(**A**) Synthesis of the peracetylated 1,7-bicylclic lactone of KDN **26** [79]; (**B**) synthesis of the protected 1,7-bicylclic lactone of KDN **29** [80].

As we will explain shortly, the bicycle lactones of Neu5Gc **4d** and KDN **4f** have been less studied than those of Neu5Ac **4a**. The first attempt to isolate and characterize these compounds, in a protected form, was realized by Ogura’s group in 1992 [79] on the peracetylated derivative of KDN **26** (Scheme 7A). The protected lactone **26** was reached, as a major compound from KDN **1c**, after acylation with acetic anhydride in pyridine and esterification, together with compounds **27a** and **27b**. The authors clarified the structure of **26** using NMR and X-ray diffraction analyses.

A second work, published in 1994 by David et al. [80], described the reaction of the partially protected Neu5Ac derivative **28** with tributyltin oxide, affording the 1,7-lactone **29** in 55% yield (Scheme 7B).

These two articles remain the only sporadic attempts of achieving 1,7-protected lactones of KDN; while there are no papers on obtaining Neu5Gc lactone derivatives, before the articles published by Allevi et al. in the 2010.

**Scheme 8:**
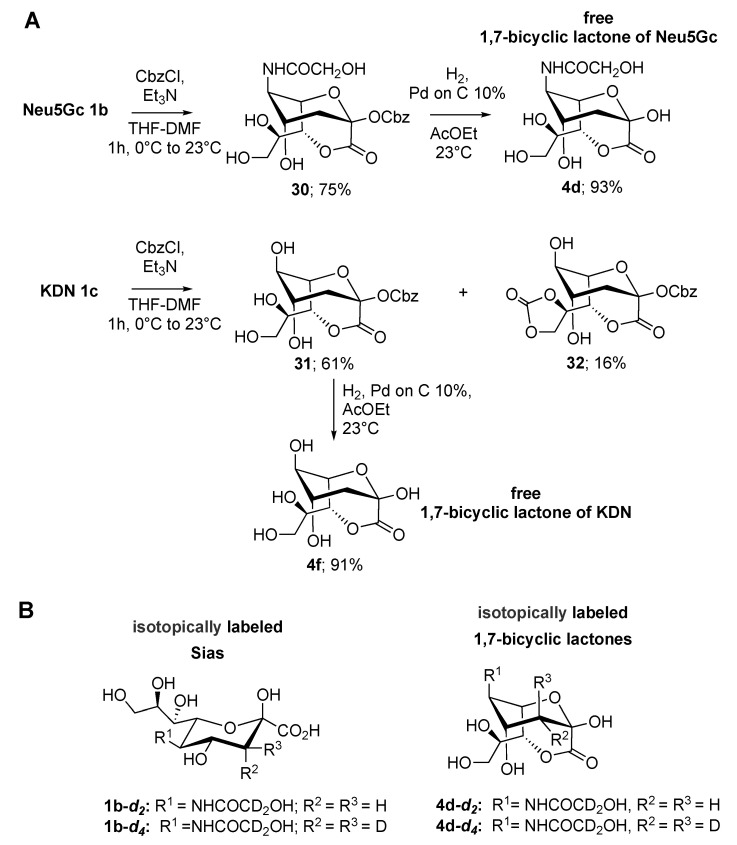
(**A**) Synthesis of the free 1,7-bicyclic lactone of Neu5Gc **4d** and of KDN **4f**, starting from Neu5Gc **1b** and KDN **1c**, respectively [19]; (**B**) isotopically labeled 1,7-byciclic lactones **4d-*d*_2_** and **4d-*d*_4_** derived from the corresponding Sias **1b-*d*_2_** and **1b-*d*_4_** [18].

In that year, Allevi et al. [19], in light of the knowledge on Neu5Ac **4a** lactone, synthesized the free 1,7-lactones of Neu5Gc **4d** and KDN **4f**. As depicted in Scheme 8A, Neu5Gc **1b** was transformed to the protected 1,7-lactone **30**, under similar reaction conditions reported for the achievement of compound **24**. Then, the hydrogenolysis of this intermediate, in anhydrous ethyl acetate, afforded the desired lactone of Neu5Gc **4d**. A similar route, carried out on the KDN **1c**, led to the formation of its protected 1,7-lactone **31**, together with the by-product **32**. Finally, the hydrogenolysis of **31** gave the free 1,7-bicyclic lactone **4f**.

Notably, the set-up of an accessible and versatile route to synthesize the 1,7-lactones **4a**, **4d**, and **4f**, in high yields, allows their use as authentic standards for analytical investigations and biological evaluations.

Moreover, the deuterated derivatives of Neu5Gc have been reported as authentic internal standards for the analysis by MS spectrometry techniques [18]. Similarly to that described above for Neu5Ac derivatives, isotopic lactones **4d-*d_2_***, **4d-*d_4_*** have been accomplished starting from the isotopically labeled Sias **1b-*d_2_***, **1b-*d_4_*** (Scheme 8B), under analogous reaction conditions to those previously described (see Section 2.1).

### 2.3. Synthesis of γ-Lactones of Sias

The free γ-lactone of Neu5Ac **33** was initially studied only as a synthetic synthon, as it had never been detected in biological samples. However, more recently, its formation was shown during the degradation of 1,7 Sias lactones, which could be present in natural matrices [78]. Historically, the synthesis of this lactone was instrumental for the attribution of the correct Neu5Ac **1a** C4 configuration [80,81,82] (for a complete discussion of this topic, see recent book chapter “Exploration of the Sialic Acid World” by R. Schauer and J. P. Kamerling) [1]. Moreover, other protected γ-lactones have been shown to be key intermediates in the synthesis of several natural compounds and enzymatic inhibitors.

**Scheme 9:**
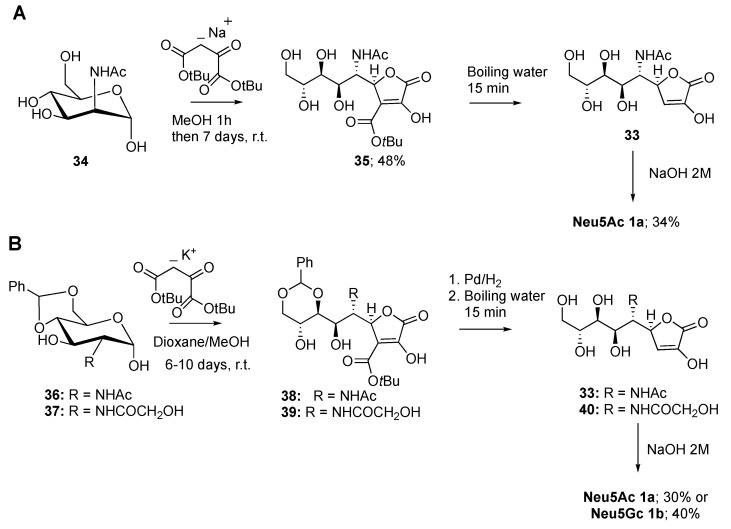
(**A**) Synthesis of Neu5Ac **1a** starting from *N*-acetylmannosamine **34** [83]; (**B**) synthesis of Neu5Ac **1a** and Neu5Gc **1b** starting from the protected compounds **36** and **37**, respectively [83,84].

Kuhn et al. led some pioneering studies in the field in the late 1950s and 1960s. In particular, in 1962, they proposed a synthetic route to Neu5Ac **1a** via the free γ-lactone of Neu5Ac **35** (Scheme 9A), which also allowed reassessment and reassignment of its stereochemistry [83]. Briefly, the condensation of the *N*-acetylmannosamine **34** and *tert*-butyloxaloacetate in methanol (MeOH) yielded the 3-carboxy-*t*-butyl γ-lactone of Neu5Ac **35** in 48% yield. Successively, lactone **35** was decarboxylated, affording the free γ-lactone **33**, which was directly hydrolyzed in 2M sodium hydroxide (NaOH) to give the pure Neu5Ac **1a** (34% yield). Comparable results were obtained starting from the 4,6-*O*-benzylidene derivative of *N*-acetylglucosamine **36**, giving the protected lactone **38**, eventually affording lactone **33** after deprotection and decarboxylation. Finally, compound **33** was directly hydrolyzed to Neu5Ac **1a** in 30% yield (Scheme 9B).

A few years later, this synthetic method was applied by Faillard for the synthesis of *N*-glycolylneuraminic acid **1b** (Neu5Gc), starting from *N*-glycolylglucosamine **37** via its protected and free γ-lactones, **39** and **40**, respectively [84]. The free γ-lactone **40** could be converted into the pure Neu5Gc **1b** under basic conditions (Scheme 9B).

The free γ-lactone of Neu5Ac was obtained also by Dereviskaya [16], although without any chemical-physical properties elucidation. In fact, the complete chemical-physical characterization (NMR, MS, ect.) of this molecule was reported only several decades later, when two independent synthetic routes were published [77,85], as discussed later in this review.

**Scheme 10:**
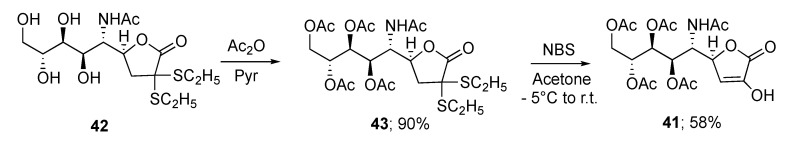
Synthesis of protected γ-lactone **41** [88].

Between 1966 and 2010, major efforts in the field were directed to study some protected γ-lactones, as they were suitable intermediates to synthesize various Neu5Ac substituted derivatives. In those years, two independent studies by Khorlin [86] and by Holmqusit [87] are of particular interest, as they described the synthesis of several sialidase inhibitors. Successively, Zbiral and Schmid obtained the acetylated γ-lactone of Neu5Ac **41** in good yield (52% overall yield) [88]. This γ-lactone was synthesized from compound **42**, via a classical acetylation reaction to afford the intermediate **43**, followed by treatment with *N*-bromosuccinimide (NBS) (Scheme 10).

**Scheme 11:**
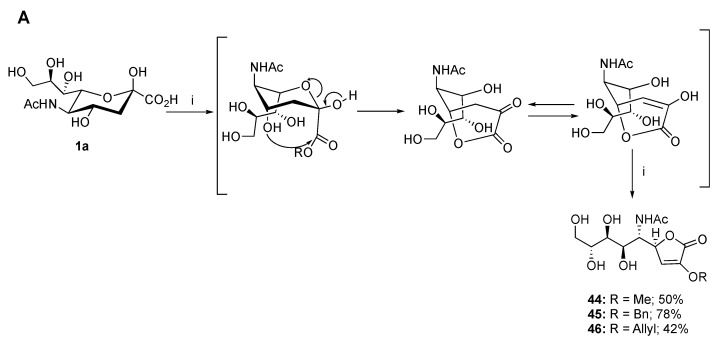

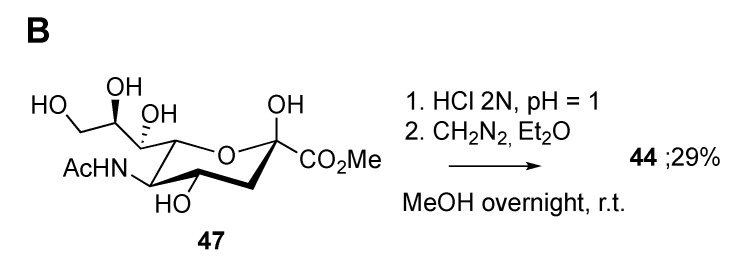
(**A**) Synthesis of protected γ-lactones **44–46** and the proposed reaction mechanism [89]; (**B**) alternative synthesis of compound **44** [70].

Very significant results in this field were reported in the 1980s by Ogura’s group [70,88], concerning the synthesis of some differently protected γ-lactones of Neu5Ac of relevant biological interest. To this purpose, Neu5Ac **1a** was first dissolved in DMF and then treated with an excess of cesium carbonate (Cs_2_CO_3_), together with the appropriate reagent: (i) methyl iodide, (ii) benzyl bromide and (iii) allyl bromide, respectively. After 1.5–3 h at 40 °C, the corresponding protected lactones **44–46** where obtained in 42–78% yield (Scheme 11A). Additionally, the methylated γ-lactone **44** could be obtained treating the methyl ester **47** in methanol solution with diazomethane (overnight at r.t.) in 29% yield (Scheme 11B) [70].

In the same studies, the authors hypothesized a complex mechanism for the formation of the γ-lactone, via a ^2^C_5_ to ^5^C_2_ conformation-switch, followed by an intramolecular ester exchange reaction with retention of the C4 stereochemistry (Scheme 11A) [89]. Remarkably, the proposed mechanism could only be experimentally confirmed twenty-seven years later, by Allevi’s group, as described below. Surprisingly, no further studies on γ-lactones were published for over two decades, with the exception of the highly efficient synthesis of KDN **1c** by Warwel in 2000 [90], which involved the free γ-lactone of KDN as key intermediate.

**Scheme 12:**
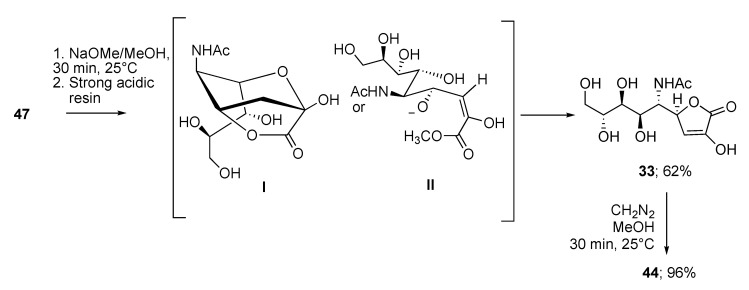
Synthesis of the free γ-lactone **33** starting from the methyl ester **47** [85].

Only in 2010 Sawada and coworkers [85] were able to isolate the free form of the γ-lactone of Neu5Ac **33** (62%), upon treatment of Neu5Ac methyl ester **47** under basic conditions using sodium methoxide (NaOMe 0.5 M in methanol for 0.5 h at 25 °C), followed by a neutralization and a final re-crystallization step (Scheme 12).

**Scheme 13:**
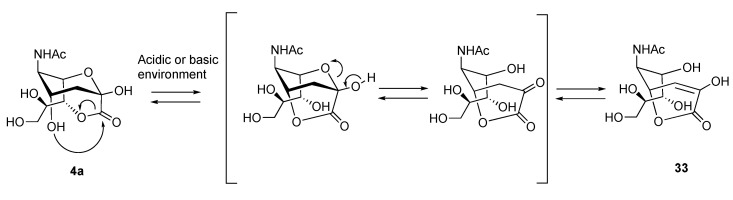
Synthesis of the free γ-lactone **33** starting from the bicyclic 1,7-lactone **4a** [78].

Curiously, two possible intermediates (**I** or **II**) involved in the reaction mechanism were proposed: a bicyclo-1,4 lactone **I** or a ring-opened intermediate structure **II** (Scheme 12). Moreover, to confirm the correct structure, the obtained γ-lactone **33** had to be transformed into the corresponding methyl ether derivative **44** by treatment with diazomethane. More recently, in 2015, Allevi’s group described a novel synthetic route to obtain the free γ-lactone **33** in very high yields (83–91%), starting from the bicyclic 1,7-lactone **4a** and using different acidic and basic reaction conditions in a definite reaction time [78] (Scheme 13) (for detail about reaction conditions, see Section 3.4).

**Scheme 14:**
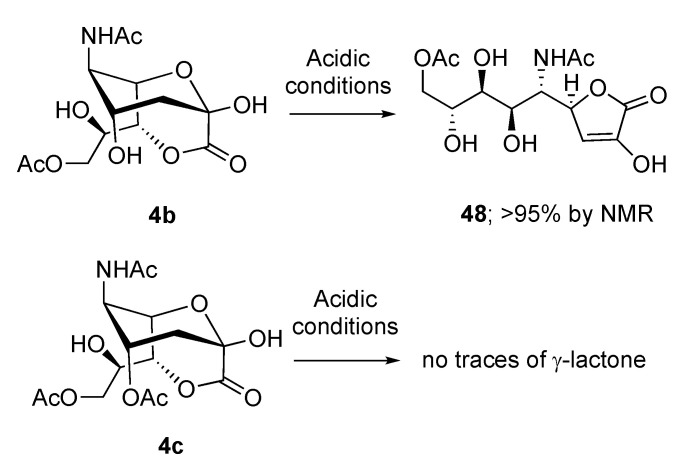
Synthesis of the protected γ-lactone **48** starting from the corresponding bicyclic 1,7-lactone **4b** [78].

Interestingly, they discovered that the bicyclic 1,7-lactone **4a** could evolve into the γ-lactone **33**. These could be rationalized, taking into account the pioneering work of Ogura [89]. In particular, the formation of the γ-lactone, from different esters of Neu5Ac, goes through an intermediate flipped structure, obtained by an inversion from a ^2^C_5_ to a ^5^C_2_ conformation, the latter being similar to that of the 1,7-lactone. In light of this unexpected rearrangement, Allevi and coworkers proposed a possible mechanism for the formation of the γ-lactone, involving the intramolecular transesterification of the free bicyclic 1,7-lactone **4a**, mediated by the attack of the C4 hydroxy group on the carboxyl function at C-1, under acidic or basic conditions (Scheme 13). According to the proposed mechanism [78], the 9-acetylated γ-lactone **48** could also easily be obtained, starting from the corresponding 1,7-lactone **4b**. Conversely, the protection of the C4 hydroxyl group, as shown in the compound **4c**, prevented the rearrangement, thus blocking the formation of the corresponding γ-lactone (Scheme 14; for detail about reaction conditions, see Section 3.4).

## 3. Biological Relevance of Neu5ac 1,7-Lactones

To date, the only intramolecular lactones identified in biological matrices, and related to physio-pathological states, are the 1,7-lactones of Sias **4a–e**. Instead, to the best of our knowledge, 1,4- or γ-lactone derivatives have not been identified in glycoconjugates.

In this section, before reviewing the biological functions of the 1,7-lactones of Sias, we will briefly touch on the analytical method used for their identification in biological samples [60]. Indeed, these two aspects are closely related, as the identification of these molecules, which has been carried out almost exclusively with the gas chromatography–mass spectrometry (GC-MS) method developed by Zanetta and collaborators in 2001 [60], could have generated incorrect results due to some recently discovered glitches in the analytical method [66]. However, recently, the use of biochemical assays (differential scanning fluorometric, kinetic studies) performed with authentic standards of these lactones has overcome the limitations of the GC-MS method by Zanetta [63,64].

### 3.1. Analytical and Biochemical Techniques Applied to Identify Neu5Ac 1,7-Lactones

**Scheme 15:**
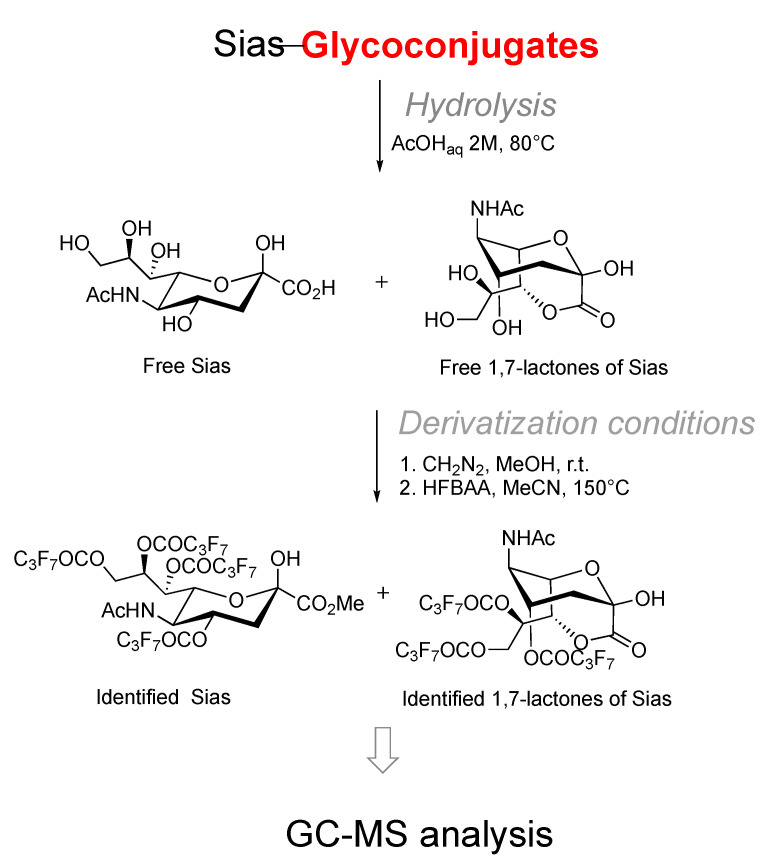
The three fundamental steps of Zanetta’s gas chromatography–mass spectrometry (GC-MS) method. From the hydrolysis of the glycoconjugates with acetic acid (AcOH), different Sias and their lactones are produced. Here, by way of example, the structures of Neu5Ac **1a** and its lactone **4a** have been reported [60].

The GC-MS method, reported by Zanetta et al. in 2001 [60], was used to identify several sialic acids derivatives in biological samples. The three fundamental steps of the method are depicted in Scheme 15, and consist of: (i) the release of sialic acids from the glycoconjugates; (ii) the derivatization of their carboxylic function with diazomethane and (iii) the derivatization of the hydroxyl functions with heptafluorobutyric anhydride (HFBAA) (except the semi-acetal one at C2, see Scheme 15 for details). It should be noted that the 1,7-lactones of Sias were the only subclass of compounds that could be determined without the derivatization step with diazomethane.

The main features of this method, which quickly became the preferred analytical method in the field, were: (i) the possibility to analyze different classes of compounds, including oligosaccharides, glycolipids and glycoproteins; (ii) the low interference of peptides and amino acids, which are present in the sample and that can be simultaneously analyzed with the Sias; (iii) the lack of intermediate purification steps of Sias after the initial hydrolysis; (iv) the prompt identification of the different analytes as heptafluorobutyrate (HFB) derivatives using their specific ions in the MS profile; (v) the stability of the HFB derivatives.

However, a critical pitfall of the analytical method was overlooked for almost two decades [60]. In fact, due to the lack of authentic reference samples for most Sias, including the 1,7-lactones, their structure was established only by the mass fragmentation profile, after a required multistep isolation and derivatization process that could alter their original structure. Indeed, it was recently shown [66] that the 1,7-bicyclic lactones, including the three derivatives of *N*-acetylneuraminic acid **4a–c** and the two derivatives of *N*-glycolylneuraminic **4d–e**, cannot be determined using the current analytical methodology (see Section 3.4) [60].

The GC-MS method of Zanetta et al. was also used in a study investigating the physiological role played by the 1,7-lactones in the interleukin-4 (IL-4) signaling modulation [59]. In particular, the authors combined the GC-MS approach with more traditional biochemical techniques, including the use of glycoconjugates immobilization methods coupled with electrophoresis, blotting techniques, and enzymatic inhibition assays using specific oligosaccharides.

### 3.2. 1,7-Lactones of Sias in Biological Samples

The GC-MS analyses performed by Zanetta et al.’s method revealed the presence of 1,7-lactones in mucins of different physiological and pathological tissues from different species.

#### 3.2.1. Animal Tissues

The 1,7-lactones of Sias have been initially detected in mucins of different animal species, such as in the bovine (1%) and ovine (0.5%) submandibular glands, in eggs of *B. bufo*, and in the skin of *A. Anguilla*. Among them, the mucins of *B. bufo* eggs were very rich in these lactones, containing all the known compounds **4a–e**, where the derivative **4a** was the most abundant [60].

Successively, the 1,7-lactone **4a** was detected in mouse tissues in a study aimed at evaluating the distribution of the different sialic acids as endogenous substrates of coronavirus hemagglutinin-esterases [50]. In particular, the authors evaluated the distribution of 13 sialic acids, including lactone **4a**, linked to glycoproteins and glycolipids at different stages of mouse brain development. They found that the 1,7-lactone **4a** was not expressed in glycoproteins of mouse embryonic tissues nor in the newborn or the adult mouse brain. On the other hand, they found modest quantities (0.35% of total Sias) of **4a** in seven-day-old mice. Additionally, they found that, among the extracerebral tissues, only the liver contained this lactone (3.54% of total Sias). In this study, the 1,7 lactone **4a** was only found in the initial embryonic state (on the thirteenth day), then it could not be detected until the mouse birth. Then, at seven days from birth, it was widely expressed (57.73% of total Sias), although its content was reduced to minor quantities (0.20% of total Sias) in the adult mouse. Curiously, a high concentration of this lactone was detected in the metencephalon of adult mouse tissue; on the other hand, it was absent in other cerebral or extracerebral compartments. Unfortunately, the biological significance of the occurrence of Neu5Ac1,7L in large quantities in the brain and in the liver is still puzzling to date [50].

#### 3.2.2. Human Healthy and Diseased Tissues

The physiological presence of the 1,7-lactone **4a** and its interaction with interleukin 4 (IL-4) in human tissues was investigated, suggesting the possible clinical use of this Neu5Ac derivative as a potential modulator of IL-4-mediated immune response. More specifically, in 2001, Zanetta’s group disclosed that the 1,7-lactone **4a** was the ligand of human interleukin 4 [56,58,59], thus proposing a “lectin” activity of IL-4. In fact, they found that this interleukin could bind to the mucins, extracted from *B. bufo* eggs, which are glycoproteins enriched in sialylated 1,7-lactones. Moreover, the authors excluded that the 1,7-lactone of Neu5Gc could be the ligand of IL-4 in the *B. bufo* mucins, as they performed analogous experiments on the mucins of the ovine submandibular gland, where this lactone is known to be absent [60]. Importantly, the 1,7-lactone **4a** could modulate the signal transduction events initiated by IL-4 via a 63 kDa protein (p63) on resting lymphocytes, supporting that the pathway depends on the lectin activity. Overall, the study suggests that specific carbohydrates, such as the 1,7-lactones of Sias, could be instrumental to modulate the immune system, as an alternative to therapies employing antibodies against interleukins or interleukin receptors. In fact, the involvement of the 1,7-lactone **4a** in immunomodulation has been also supported by some studies performed on human purified glycoproteins from pregnant women [53]. Specifically, this molecule is highly expressed in both the mucin MUC2, having a high degree of *O*-linked glycans, and the uromodulin, holding essentially *N*-linked ones. Interestingly, the 1,7-lactone **4a** accounted for 7.7% and 35% of the overall sialic acids present in MUC2 and in the uromodulin protein, respectively. Indeed, the urine glycoproteins of pregnant women are known to specifically bind IL-4 in a Sias carbohydrate-dependent way, although the actual ligand(s) have yet to be identified [56,58,59]. Thus, these results [53] seem to support that lactone **4a** could be the ligand of IL-4, although further studies to confirm this hypothesis are still needed.

The different expression of 1,7-lactone in pathological and healthy human tissues gave a particular relevance to this molecule, suggesting its possible role as a biomarker. For example, a high amount of 1,7-Neu5Ac lactone **4a** has been detected in glycoproteins derived from the human colon-rectum tissues, supporting its use as a possible biomarker of colon cancer, if overexpressed in pathological tissues [53]. Moreover, the presence of Neu5ac1,7L in mucins and oligosaccharides isolated from the human gut has also been reported [55]. The study underlined the difference in glycosylation of distinctive parts of the intestine. Consistent levels of lactone **4a** were found in different gut compartments, the highest being found in the ileum (20–21% of total sialic acids in each sample) and in the rectum (19–26%), whereas minor quantities were detected in the cecum (11–20%), in the transverse (8–14%), and in the sigmoid (6–9%).

Interestingly, the GC-MS analytical method of Zanetta et al. was applied to evaluate human erythrocyte membrane glycoprotein- and glycolipid-containing sialic acids of different serotypes [54]. Remarkably, these lactones were absent from the red blood cell (RBC) samples of healthy people, but were present, sometimes even at a high level, in several malignant tumors. In 2007, the Neu5Ac 1,7-lactone **4a** was found in the erythrocyte membranes of patients affected by polycythemia vera [49]. In particular, a drastic reduction in the Sias diversity was observed, as well as the absence of Neu5Gc and its derivatives. Despite that, the 1,7-lactone **4a** became a predominant compound, suggesting a crucial role of this molecule to discriminate between healthy and diseased RBC samples. Furthermore, in a study on malignant melanocytes [65], lactone **4a** was found as a minor component in healthy tissues, but the dominant Sia in some malignant cells. Specifically, it was found that the Sia of monosialodihexosylganglioside (GM3) is mainly in the form of the internal 1,7-lactone **4a**. Based on the known interaction between IL-4 and 1,7-lactone, the authors proposed that the high expression of GM3 in malignant melanocytes was instrumental to “disturb” the activity of the immune system near the tumors [65].

### 3.3. Recent Insights into the Biological Role of Neu5Ac 1,7-Lactone

The biological role of **4a** has been recently investigated in the colonization of the human intestine by commensal bacteria, such as *E. coli* [63,64]. In fact, these 1,7-lactones are relatively common in the large intestine, supporting the role of this molecule as an essential source of bacterial nutrition. In this regard, a recent work by Horne et al. [63] investigated the role of the *E. coli* YjhC enzyme, and its involvement in sialic acid catabolism. Notably, differential scanning fluorimetry experiments, coupled with Phenotype MicroArray, revealed that *N*-acetylneuraminic acid **1a** and its 1,7-lactone **4a**, along with reduced nicotinamide adenine dinucleotide (NADH), could be the biologically relevant substrates of YjhC. Notably, the 1,7-lactone **4a** was selected as a model compound for in silico docking experiments, based on the obtained crystal structure of YjhC, to support the binding of this molecule in the enzyme catalytic pocket.

### 3.4. Stability of the Lactones of Biological Interest and Questioning on the Real Presence of 1,7-Lactones of Sias in Biological Tissues

**Scheme 16:**
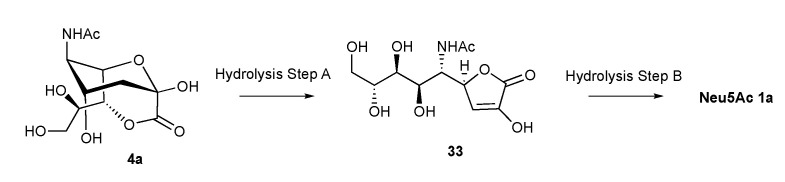
General two-steps hydrolysis of compound **4a** (see Table 1 for reaction conditions) [78].

As outlined in this review, the 1,7-lactones of Neu5Ac and Neu5Gc have been shown to play important roles both physiologically and pathologically. However, the relative stability of these compounds is a key feature that needs to be addressed, especially when assessing them in biological samples using multi-step extraction and purification procedures. In this regard, during their synthesis in free form (see Section 2.1 and Section 2.2) [17,18,19,20,21,66,78], Allevi’s group clarified significant features regarding the stability of these molecules. In particular, lactone **4a** was found to be unstable in protic solvents, such as methanol and water, eventually affording the corresponding Neu5Ac methyl ester or the free Neu5Ac [66,78]. The same authors also elucidated the behavior of these molecules in different acidic and basic environments. In particular, treatment with a strong acidic resin (for 1 h at r.t.) led to the formation of the γ-lactone **33**, which was transformed in free Neu5Ac **1a**, after some additional time (i.e., 120 h at 25 °C or 8 h at 80 °C—Scheme 16 and Table 1, entries 1–2). Analogous results were obtained after treatment with hydrochloric acid (HCl) 1M for 1 h at 80 °C of (Entry 3). Alternatively, treatment of **4a** for 24h at RT with a weak acid, such as AcOH 2M, led to the complete transformation into the γ-lactone **33** (Entry 4). A similar behavior was observed when the stability of **4a** was tested under basic conditions. In particular, treatment with NaOMe for 30 min at 30 °C led to the formation of the γ-lactone **33** in very high yields (92%, entry 6). Similarly, the use of 1,1,3,3-tetramethylguanidine (TMG) 0.05 M at 25 °C for 9 min afforded the same compound, which was quantitatively transformed into Neu5Ac (Entry 7 and 8), after an additional time of 72 h at 25 °C (or 3h at 60 °C).

**Scheme 17:**
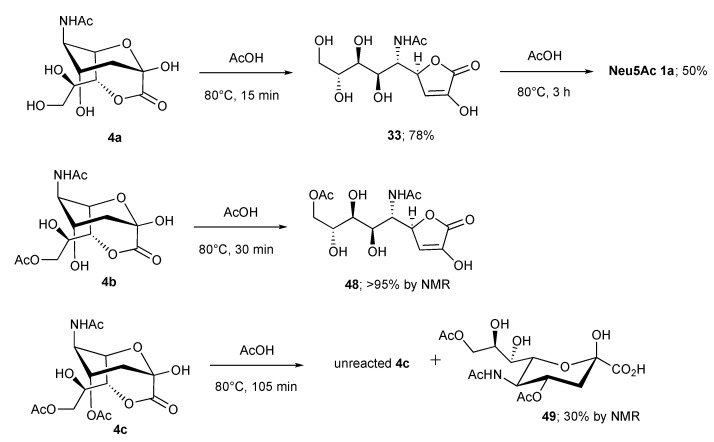
Reactivity of 1,7-lactones **4a**, **4b** and **4c** under acidic conditions (AcOH, 80 °C) [78].

Surprisingly, when the authors investigated the standard hydrolytic conditions used to cleave Sias derivatives from glycoconjugates [60], they found that treatment of **4a** with AcOH 2M at 80 °C for 15 min yielded exclusively the γ-lactone **33** (entry 5 and Scheme 17), which eventually evolved in Neu5Ac **1a** after an additional time of 3 h. Similarly, the C9 monoacetylated 1,7-lactone **4b**, after 30 min at 80 °C in AcOH 2M, rapidly evolved in the corresponding γ-lactone **48** (>95% yield, quantification by NMR) [78]. On the other hand, the C4, C9 diacetylated 1,7-lactone **4c** was stable under these conditions and, only after 105 min, a partial degradation occurred, which was accompanied by the formation of the sialic acid derivative **49** (around 30% yield, quantification by NMR) (Scheme 17).

Overall, these results support that these 1,7-lactones could not survive under the acidic hydrolysis conditions required for their cleavage from glycoconjugates in Zanetta’s method [60], calling for a complete reevaluation of their presence in biological samples and of their physiological and pathological roles.

**Scheme 18:**
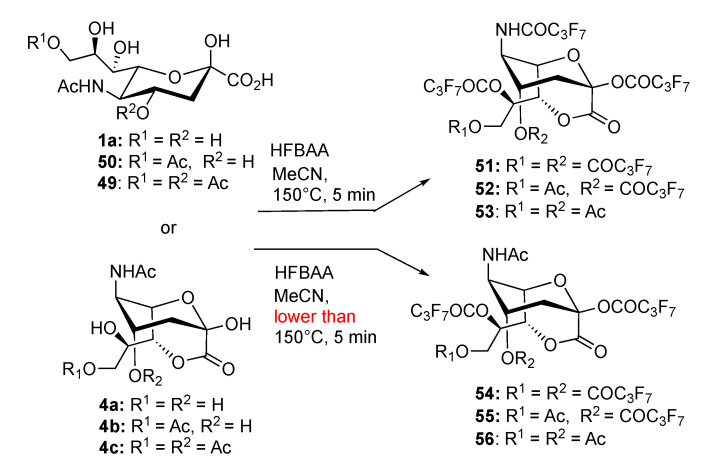
Derivatization conditions with HFBAA explored by Allevi’s group [66].

Moreover, in the same article, Allevi’s group clarified some critical flaws of the GC-MS analytical protocol used to detect Sias, including the 1,7-lactonized derivatives, as heptafluorobutyrates [66]. Performing GC–MS and NMR analyses, using the authentic reference standards of free Sias (Neu5Ac **1a**; 9-acetyl Neu5Ac **50**; and the 4,9-diacetyl Neu5Ac **49** derivatives) and their 1,7-lactonized analogues (**4a, 4b** and **4c**), they discovered interesting features of these compounds (Scheme 18).

In particular, they demonstrated [66] that, under the reported conditions, all the sialic acid derivatives had been converted to 1,7-lactonized *N*- and *O*-heptafluorobutyrates compounds, and not the expected methyl esters. Indeed, treating Sias (**1a**; **49**; **50**) or their 1,7-lactones (**4a**; **4b**; **4c**) with this heptafluorobutyric anhydride in MeCN for 5 min at 150 °C (the derivatization conditions commonly used in literature [60]), only the *N*-transacylated [91,92,93,94] heptafluorobutyric lactones **51–53** were obtained. Moreover, they established that the correct structures of the supposed 1,7-lactones present in the physiological media, and overexpressed in different pathologies, are those of lactones **54–56**. Indeed, compounds **54–56**, formed by incorrect derivatization (low temperature or low reaction times), showed the same MS fragmentation profiles previously found for 1,7-lactones by Zanetta’s GC-MS method [49,50,51,52,53,54,55,56,59,65].

Overall, they substantiated that 1,7-lactones of Sias, if present in biological samples, decompose under the acidic hydrolysis conditions used for their cleavage and they are accidentally generated as unexpected artifacts during the derivatizing step from their homologs acids [66].

## 4. Conclusions and Future Perspectives

As detailed in this review, the internal lactones of sialic acids play a central role in many physiological and pathological processes. However, recent advances in understanding their reactivity and stability have also exposed the limits of their identification and quantification from biological samples [66]. Clearly, milder hydrolytic strategies are needed to remove Sias from glycoconjugates and overcome the lability of 1,7-lactone derivatives in free form. This, together with the recent advances in the synthesis of authentic standard samples, could allow the development of new analytical methods to confirm their presence in biological matrices, eventually reassessing their physiological and pathological roles.

## Figures and Tables

**Table 1:** Hydrolysis reaction conditions of compound **4a** (Step A) and compound **33** (Step B) [78].

Entry	Reagent	T (°C)	Step A		Step B	
			t (h)	Yield (%) of 33	t (h)	Yield (%) of 1a
entry 1	Dowex H^+ a^	25	1	86	120	87
entry 2	Dowex H^+ a^	80	-	-	8	81
entry 3	1M HCl ^a^	80	-	-	1	65
entry 4	2M AcOH ^a^	25	24	83	-	-
entry 5	2M AcOH ^a^	80	0.25	78	3	50
entry 6	0.5M NaOMe ^b^	30	0.5	92	-	-
entry 7	0.05M TMG ^b^	25	0.15	91	72	78
entry 8	0.05M TMG ^b^	60	-	-	3	97

^a^ solvent H_2_O ^b^ solvent MeOH.

## Abbreviations

AcOH	Acetic acid
CMP	Cytidine mononucleotide
Cs_2_CO_3_	Cesium carbonate
DANA	2-Deoxy-2,3-dehydro *N*-acetylneuraminic acid
DCC	*N*,*N*′-dicyclohexylcarbodiimide
DMF	Dimethylformamide
GC	Gas chromatography
GC-MS	Gas chromatography–mass spectrometry
GM_3_	Monosialodihexosylganglioside
HCl	Hydrochloric acid
HFB	Heptafluorobutyrate
HFBAA	heptafluorobutyric anhydride
HPLC	High-performance liquid chromatography
IL-4	Interleukin 4
kDa	Kilodalton
KDN	3-Deoxy-D-*glycero*-D-*galacto*-2-nonulosonic acid or 2-keto-3-deoxy-D-*glycero*-D-*galacto*-nononic acid
MeCN	Acetonitrile
MeOH	Methanol
MS	Mass spectrometry
NADH	Nicotinamide adenine dinucleotide, reduced
NaOH	Sodium hydroxide
NaOMe	Sodium methoxide
NBS	*N*-Bromosuccinimide
Neu	5-Amino-3,5-dideoxy-D-*glycero*-D-*galacto*-2-nonulosonic acid or Neuraminic acid
Neu5Ac	*N*-acetylneuraminic acid
Neu5Ac1,7L	*N*-acetylneuraminic acid 1,7-lactone
Neu5Gc	*N*-glycolylneuraminic acid
NMR	Nuclear magnetic resonance
RBC	Red blood cell
Sias	Sialic acids
THF	Tetrahydrofuran
TMG	1,1,3,3–Tetramethylguanidine
